# Effects of Pine Needle Extracts on the Degradation of LLDPE

**DOI:** 10.3390/polym15010032

**Published:** 2022-12-22

**Authors:** Xiangyao Li, Jie Zhang, Chengchao Liu, Wenmin Mu, Zhe Kong, Yan Li, Zhongwei Wang, Qing Yu, Guiqing Cheng, Long Chen

**Affiliations:** 1 College of Materials Science and Engineering, Shandong University of Science and Technology, Qingdao 266590, China; 2 School of Biological and Chemical Engineering, Qingdao Technical College, Qingdao 266555, China

**Keywords:** natural, antioxidant, polyethylene, application

## Abstract

Polyolefin suffers from degradation during processing and application. To prolong the service life, antioxidants are needed in the packing formula of polyolefin products. The usage of natural antioxidants could avoid potential health hazards aroused by synthetic ones. Pine needles have long lives and hardly rot, suggesting their high resistance to degradation. To provide a new candidate of natural antioxidants and add more value to pine needles, pine needle extracts (PNE) were investigated as the antioxidant of linear low-density polyethylene (LLDPE). PNE-modified LLDPE (PE-PNE) exhibited much better short-term and long-term aging resistance than pure LLDPE (PE): Oxidation induction time (OIT) of PE-PNE was 52 times higher than that of PE, and the increments of carbonyl index (CI) of PE-PNE-1st samples placed under daylight and in the dark were approximately 75% and 63% of PE under the same conditions. It could be attributed to the attractive antioxidant capacity of PNE (IC_50_ of DPPH radical scavenging was 115 μg/mL). In addition, the PE-PNE sample showed high processing stability and maintenance of the mechanical property during multiple extrusions: only a 0.2 g/10 min decrease in melting flow rate was found after five extrusions; the tensile strength and elongation at break were almost unchanged. All results reveal that pine needle extracts could play a role in LLDPE stabilization. Moreover, as pine needles are mainly considered a kind of waste, the present study would benefit the budget-reducing polyolefin industry.

## 1. Introduction

Polyolefin is the most important type of plastic, with non-toxic, tasteless, good chemical resistance, easy processing, and other advantages. The usage of polyethylene (PE) and polypropylene (PP) is increasing year by year [1,2,3]. During the usage of PE products, vinyl groups in the main chain react easily with oxygen and alkyl radicals [2,4,5,6]. The latter reaction forms long-branched chains, leading to the easy degradation of PE by high temperature, shear forces, and oxygen. Such degradations of polymer chains usually cause deterioration of properties. Therefore, multiple stabilizers must be added to preserve the properties of polyolefin. There are two kinds of polymer stabilizers: primary and secondary ones [7,8,9]. Primary antioxidants inhibit oxidation by providing a hydrogen atom. Secondary antioxidants break down the peroxides formed in the oxidation process and thereby protect the primary antioxidants [7]. Such combinations are proved to be very effective and economical. However, about a decade ago, questions were raised about the potential health hazards associated with the usage of synthetic phenolic antioxidants [2,8,9]. These questions are still unsolved yet.

It is known that natural plants rot, and their decay rate is very slow. Hence, they contain certain substances that can slow down the degradation process [10,11]. Therefore, using natural plants as the raw material for antioxidants is believed to be a reliable option for inhibiting the degradation of polyolefin and solving the problems caused by synthetic phenolic antioxidants. So far, many natural substances have been reported to be promising candidates for polymer stabilizers [2,4,8]. For example, carotenoids were found to have the ability to react with alkoxy and peroxy groups and eliminate free radicals [12,13]. Natural polyphenols (including flavonoids, other polyphenol compounds, phenolic acids, phenolic polymers, etc.) could eliminate free radicals by dehyrating phenolic hydroxyl groups [14,15,16,17].

Although there are many studies on natural antioxidants, most of them are focused on the roots, stems, leaves, and fruits of plants; little attention has been paid to the usage of waste. Cerruti et al. investigated the usage of grape seeds, and positive effects on the thermal stability of PP were found [12]. Messori et al. compared the performance of solid wine wastes (peels, seeds, and stalks) in stabilizing PP [16]. With the addition of wine wastes (6 wt% of PP), the thermal stabilities of the polymer were effectively enhanced. Hazelnut skin and cocoa by-products were reported to be the antioxidant and thermal stabilizers for PP, respectively [18]. Antioxidant-rich agro-wastes (such as grape pomace waste, turmeric shavings and waste, coffee grounds, and orange peel waste) were found to be the thermo-oxidative stabilizers for low-density polyethylene (LDPE) [19]. Similarly, in our previous studies, Punica granatum peels and bamboo leaves were discovered to be efficient antioxidants for polyolefin [20,21]. Even so, there is still a long way to solve the questions raised by the usage of synthetic phenolic antioxidants. More promising antioxidants need to be discovered to enhance the stability of polyolefin in an environmentally friendly and cost-effective way without damaging their mechanical properties. 

According to our previous studies, puerarin and forsythia suspensa (traditional Chinese medicine) extracts could act as efficient stabilizers for polyolefin [22,23]. It is known that traditional Chinese medicine has a wide range [24]. Could any other ones have similar properties? Pine needles, also known as bristle pine leaves and pine hairs, are the leaves of pine plants in the Pinaceae [25]. It is found that pine needles have remarkable pharmacological effects (such as lowering blood sugar, regulating blood lipids, inhibiting tumors, and anti-aging) [26,27,28,29]. It is also well-known that both fresh and fallen pine needles hardly rot. We suspect that this phenomenon is due to the antioxidants inside pine needles. Combined with the reported medical effect, we believe that pine needles could play a role in the antioxidant modification of LLDPE. If true, more values could be added to pine needles besides returning to the soil. In addition, the usage of such waste would be a benefit for the polyolefin industry. To accomplish such objectives, low-density linear polyethylene (LLDPE) was chosen as the polymer matrix in this study. Pine needles were extracted with ethanol aqueous solution, and the performance of pine needle extracts (PNE) in stabilizing LLDPE was investigated in terms of short-term/long-term aging resistance and mechanical properties. Possible roles of PNE against the degradation of LLDPE were proposed.

## 2. Materials and Methods

### 2.1. Materials and Instruments

Materials: The powder of linear low-density polyethylene (LLDPE, MFR = 4 g/10 min) was purchased from Maoming Petro-Chemical Co., Ltd (Maoming, China). Pine needles were naturally shed needles of Japanese loch pine grown inside the campus of Shandong University of Science and Technology. Calcium stearate (CaSt) was purchased from Shanghai Aladdin Bio-Chem Technology Co., Ltd (Shanghai, China). DPPH (HPLC grade) was from Shanghai Macklin Biochemical Co., Ltd (Shanghai, China). Synthetic antioxidant 1076 was from Qingdao Truelight functional Material Technology Co., Ltd (Qingdao, China).

Instruments: twin-screw extruder (HT-35, Nanjing Rubber and Plastic Machinery Factory Co. Ltd., Nanjing, China), melt index meter (XNR-400AM, Dongguan Xihua Testing Instrument Co., Ltd., Dongguan, China), mass spectrometer (Mircomass Q-TOF Micro, Waters (Shanghai) Technology Co., Ltd., Shanghai, China), DSC tester (DSC-100, Nanjing Dazhan Testing Instrument Co., Ltd., Nanjing, China), Fourier infrared spectrometer (IS10, Symerfeld, Springfield, MO, USA), universal material testing machine (AI—7000M, Ningbo Yinzhou Jinrui Instrument Equipment Co., Ltd., Ningbo, China).

### 2.2. Sample Preparation

Extractions: Pine needles were dried and ground into powder. Certain amounts of pine needle powders were added to the Soxhlet apparatus and then extracted with ethanol aqueous solution for 1 h (70 wt% in water, the weight ratio of ethanol to pine needle powder was 1:10). After filtration, the supernatant was collected, and the precipitate was extracted again with ethanol aqueous solution. With three times extraction, the collected supernatants were mixed and directly used for the modification of LLDPE.

Modification: The powder of LLDPE was added into the obtained solution of pine needle extracts (the content of pine needle extracts was set as 2 wt% of LLDPE) and stirred at room temperature for 2 h. Before the first extrusion, the suspension of LLDPE and pine needle extracts was dried, and then CaSt (0.2 wt% of LLDPE) was added. The mixture was extruded by a twin-screw extruder at a speed of 30 rpm (the screw temperature profile from hopper to mold is 155 °C, 180 °C, 190 °C, 200 °C, 200 °C, 195 °C, 195 °C, 195 °C, 190 °C, 175 °C). Five extrusions were carried out under the same conditions. Pine needle extracts modified LLDPE samples were named PE-PNE for short. LLDPE with only CaSt and LLDPE modified with 1076 (0.1 wt% of LLDPE) were also extruded for comparison, named PE and PE-1076, respectively.

### 2.3. Characterizations

**Scheme 1:**
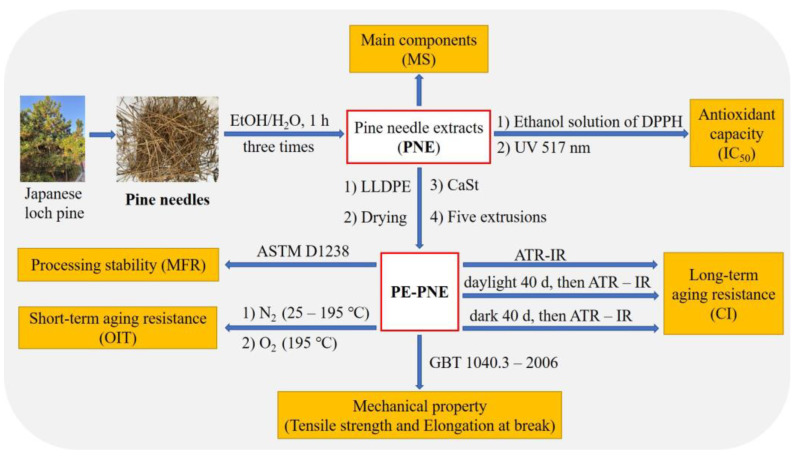
Diagram of preparation and characterizations of PE-PNE sample.

Scheme 1 depicted the diagram of preparation and characterizations of the PE-PNE sample. The same characterizations were carried out for PE, too. Detailed characterization procedures were as follows:

Mass spectrometry analysis (MS): After three extraction times, the collected supernatants were mixed and dried. The dried solids were used for the MS test with chloroform as the solvent.

Antioxidant capacity: DPPH radical scavenging tests were carried out according to the literature [30]. Typically, 0.2 mL ethanol solution of PNE was mixed with 4 mL DPPH (ethanol solution, 40 mg/mL). Then the mixture was placed in the dark at room temperature for 30 min. The area of the absorption band at 517 nm was calculated, and different amounts of PNE were used to gain the value of IC_50_.

Short-term aging resistance: Oxidation induction time (OIT) was measured by differential scanning calorimetry (DSC) at 195 °C according to our previous study [21]. Typically, the sample was heated from room temperature to 195 °C (N_2_, 20 °C/min) and then maintained for 5 min. Then the flowing phase was changed to oxygen for measuring OIT. Five tests were carried out for each sample, and the OIT values in this paper are the average data. 

Long-term aging resistance: Carbonyl index (CI) was used to characterize the long-term aging resistance, according to the ratio of band area at 1550–1780 cm^−1^ (carbonyl group) and 2850–2920 cm^−1^ (C-H band of methylene groups) [21]. Thin films were used for the tests. Original CI values of samples were obtained with samples directly taken after hot compression. PE and PE-PNE samples were placed in the same space under daylight or in the dark for 40 days. Similar tests were carried out on the samples after 40 days to gain the CI value of long-term aging. Five tests were carried out for each sample, and the CI values in this paper are the average data.

Processing stability: Melt flow rate (MFR) of the samples was tested with an MFR instrument (XNR-400 AM, Dongguan Xihua Testing Instrument Co., LTD, Dongguan, China) at 190 °C and a 5 kg load according to ASTM D 1238 standard. The MFR value is the average of five tests.

Mechanical property: Samples taken after the first, third, and fifth extrusions were tested according to GBT1040.3-2006 standard. The test object was injected with a dumbbell spline of 3 mm thickness by the injection molding machine. Tensile experiments were carried out at the rate of 20 mm/s on a universal mechanical testing machine. Five splines were tested for each formula, and the average value was taken for the tensile strength and elongation at the break of the samples.

## 3. Results and Discussion

### 3.1. Main Components and Antioxidant Capacity of Pine Needle Extracts

**Figure 1:**
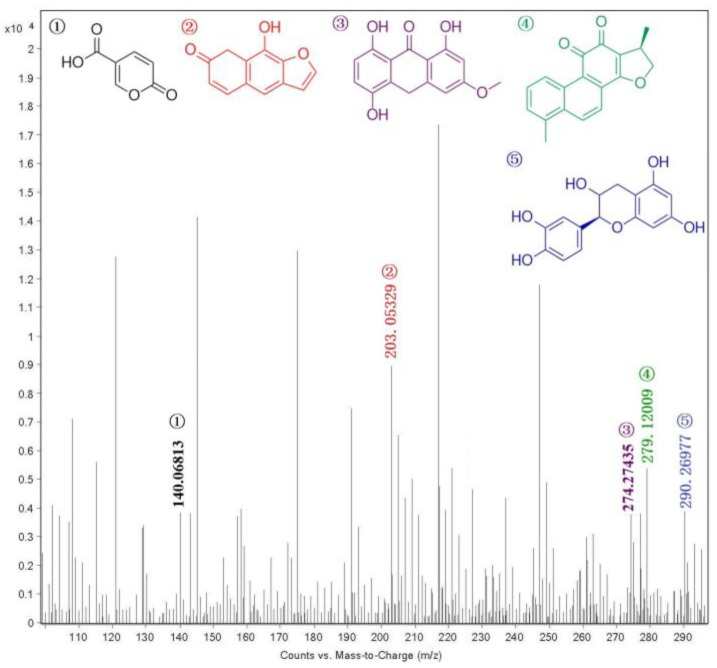
Mass spectrometry analysis of pine needle extracts. (① coumaric acid, ② xanthotoxol, ③ bellidifolin, ④ dihydrotanshinone, ⑤ catechin).

Before the application of pine needle extracts (PNE) in LLDPE modification, the main components of PNE were tested by mass spectrometry. As shown in Figure 1, catechin and bellidifolin with the typical structure of polyphenols were detected. It is known that polyphenols are common antioxidants for polyolefin [2,30,31]. Therefore, the existence of these two compounds suggests that the obtained pine needle extracts will have the possibility to act as an antioxidant for LLDPE.

Besides the two phenols, dihydrotanshinone, xanthotoxol, and coumaric acid were also detected (Figure 1). Dihydrotanshinone was reported to have the ability to protect cardiomyocytes through preconditioning regulation and antioxidant activity [30]. Xanthotoxol and coumaric acid could scavenge the DPPH radical [23,32,33]. To further certify the antioxidant capacity of PNE, DPPH radical scavenging tests were carried out. Results showed that the IC_50_ of PNE was 115 μg/mL. Although the antioxidant capacity of PNE is not as good as synthetic antioxidant 1076 (IC_50_ = 47.6 μg/mL), its capacity is among the top 15 of 100 medicinal plants investigated by Qasim et al. [34]. Thus, in order to eliminate the harmful effects of synthetic antioxidants and provide additional values of pine needles, the modification of LLDPE with PNE was carried out.

### 3.2. Effect of Pine Needle Extracts on Short-Term Aging Resistance

**Figure 2:**
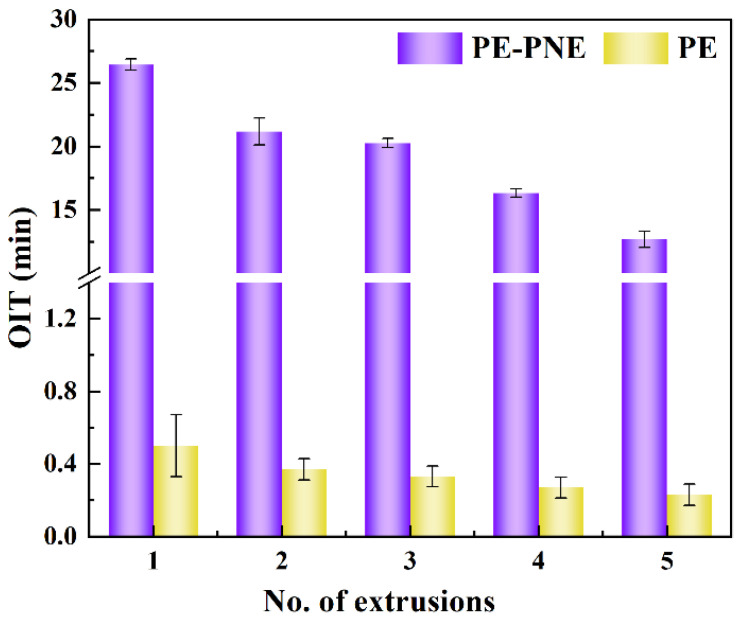
OIT values of PE and PE-PNE samples vs. the number of extrusions.

Figure 2 shows the OIT values of PE and PE-PNE samples during five extrusions. PE has almost no resistance to thermo-oxidation: the OIT values at 195 °C are below 1 min. After modification with pine needle extracts, the OIT value of PE-PNE significantly increased: it is approximately 52 times higher than PE after the first extrusion. It reveals the high ability of pine needle extracts to protect the LLDPE matrix against thermo-oxidation. Under the same conditions, LLDPE modified with 1076 has the OIT value of approximately 20 min. The results reveal that PE-PNE has similar resistance as PE-1076. Although the amount of PNE is more than 1076, the result still means a lot to the solution of harmful effects aroused by synthetic antioxidants. The OIT value of PE-PNE after the fifth extrusion is still much higher (approximately 25 times) than the PE of the first extrusion. It further illustrates the ability of pine needle extracts on the short-term aging resistance of LLDPE.

### 3.3. Effects of Pine Needle Extracts on Long-Term Aging Resistance

**Figure 3:**
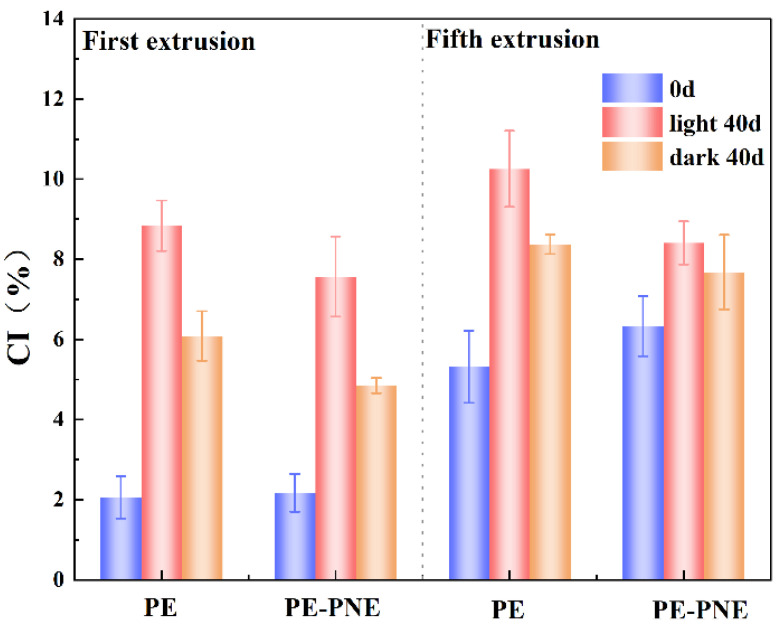
Carbonyl index (CI) of PE-PNE vs. extrusion and storage.

**Scheme 2:**
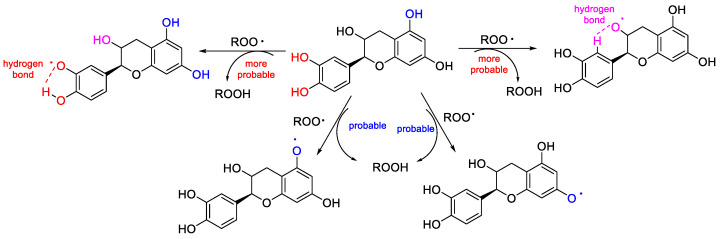
Possible role of Catechin during thermo-oxidation.

To further evaluate the protective ability of PNE against the degradation of LLDPE, the carbonyl index (CI) was employed to characterize the long-term aging degree of the material. As shown in Figure 3 and Table 1, the increments of CI values (ΔCI) of PE-PNE placed under daylight and in the dark were all lower than those of PE under the same conditions: For example, the ΔCI values of PE-PNE taken after the first extrusion were 248.8% and 123.5% for the daylight and dark cases, respectively. While the ones of PE were 329.1% and 195.6%, respectively. These results indicate that pine needle extracts could protect the LLDPE matrix against degradations caused by sunlight and oxygen in the atmosphere. Combining the results obtained with short-term and long-term aging tests, it could be concluded that pine needle extracts are a probable candidate for the substitution of synthetic antioxidants with natural ones. The protective ability of PNE towards thermo-oxidative degradation of LLDPE is consistent with the results of DPPH scavenging tests and might be related to the five compounds detected with MS (Figure 1). During thermo-oxidation, the two polyphenols could act as the H-donors, converting peroxyl radicals into hydroperoxides (less reactive) and decreasing the rate of degradation (Scheme 2). Xanthotoxol and coumaric acid are two types of lactones. Lactones could react with peroxy radicals (act as chain-breaking antioxidants), and their efficiency is considerably higher in the presence of hindered phenols [35]. Thus, the above-mentioned compounds work together to inhibit the thermo-oxidative degradation of LLDPE.

### 3.4. Effects of Pine Needle Extract on Processing Stability

**Figure 4:**
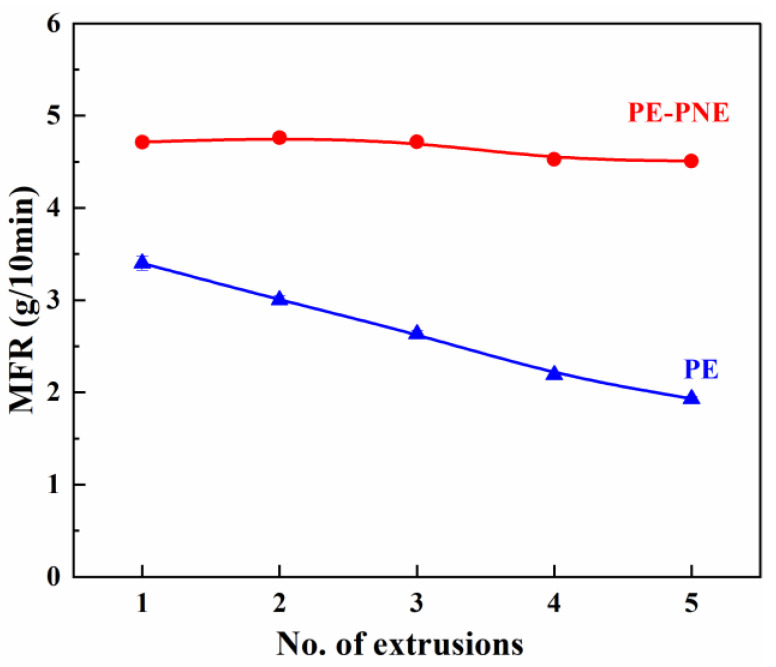
MFR values of PE and PE-PNE samples vs. the number of extrusions.

Although pine needle extracts have been proven to be an efficient stabilizer against thermo-oxidation, the amount of PNE is still higher than synthetic antioxidants. Thus, the influence of PNE to the processing of LLDPE needs to be investigated. MFR of samples is used to characterize the processing stability. As shown in Figure 4, with the increase of extrusion times, the MFR values of PE decreased significantly from 3.4 to 1.8 g/10 min (almost 50% decrement), suggesting the increment of viscosity. The decline of MFR values also reveals that PE samples underwent severe degradation during processing. A similar trend was found in Pukánszky et al. and Torkelson et al., which was usually due to structural changes, such as the formation of long chain branches and the occurrence of cross-link [5,7,19]. In contrast, the MFR values of PE-PNE samples are almost unchanged (the drop is only approximately 0.2 g/10 min), revealing the good processing stability of PE-PNE. It could be associated with the ability of antioxidants to prevent the formation of long-chain branches [5,7]. Such attractive processing stability of PE-PNE is benefit for multiple extrusions and the recycling of polymeric products, leading to certain advantages in industrial applications. Taking account of the great decline of the MFR value of PE, the above result indicates that pine needle extracts have an excellent stabilization effect against the degradation caused by heat and shear force. 

### 3.5. Mechanical Properties

**Figure 5:**
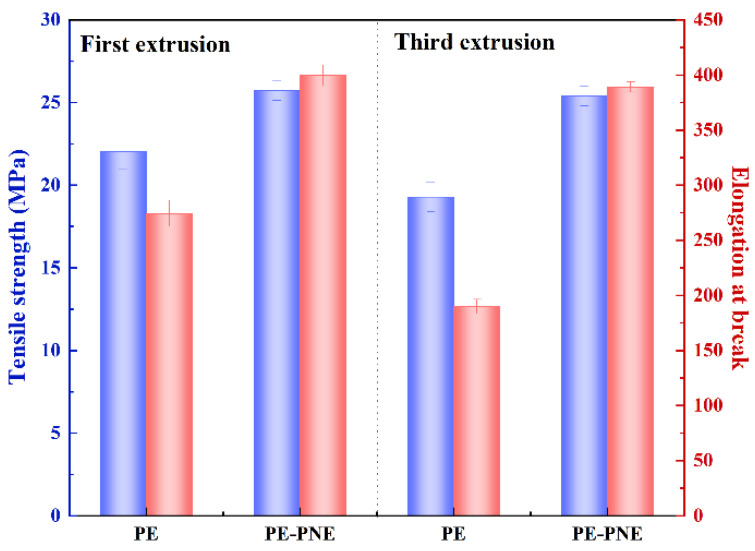
Tensile strength and Elongation at break of PE and PE-PNE samples vs. the number of extrusions.

It is known that the mechanical properties of polymers often decrease after modification due to the compatibility of additives and polymers. Although the amount of PNE is higher than synthetic antioxidants, it enhanced the processing stability. Thus, it is curious about the effect of PNE on the mechanical properties of LLDPE. As shown in Figure 5, the elongation at break of PE decreased with the increasing extrusion times. Such decline is associated with the structural change of PE due to degradation [19]. In contrast, the tensile strength and elongation at break of PE-PNE could be maintained with multiple extrusions. It means that PNE could stabilize LLDPE against degradation during multiple extrusions. Similar effects were found when antioxidant-rich waste materials were used in LDPE [19]. This result, together with the above ones, reveals that pine needle extracts could be used as the stabilizer for LLDPE without inhibiting mechanical properties. Accordingly, LLDPE modified with PNE could be applied in functional packing material with a decreased budget in the future.

## 4. Conclusions

In this paper, the properties of pine needle extract-modified LLDPE were compared with pure ones. Short-time and long-time aging results reveal that pine needle extracts could protect the LLDPE matrix against heat, oxygen, and sunlight. MFR results suggested the positive effect of pine needle extracts on the inhibition of degradation caused by heat and shear force. All results suggest pine needle extracts are efficient and multifunctional stabilizers for LLDPE. Since the addition of pine needle extracts has little influence on the mechanical properties of LLDPE, the modification in this study has potential market applications.

## Figures and Tables

**Table 1:** The increments of carbonyl index values (ΔCI) of PE and PE-PNE samples after long-term storage (40 days) under daylight or in the dark.

Samples	First Extrusion		Fifth Extrusion	
	PE	PE-PNE	PE	PE-PNE
ΔCI (under daylight, %)	329.1	248.8	92.8	32.8
ΔCI (in the dark, %)	195.6	123.5	57.3	21.3

## Data Availability

The data presented in this study are available on request from the corresponding author.
